# Acknowledgement to Reviewers of *Pathogens* in 2019

**DOI:** 10.3390/pathogens9010061

**Published:** 2020-01-15

**Authors:** Pathogens Editorial Office

**Affiliations:** MDPI, St. Alban-Anlage 66, 4052 Basel, Switzerland

The editorial team greatly appreciates the reviewers who have dedicated their considerable time and expertise to the journal’s rigorous editorial process over the past 12 months, regardless of whether the papers are finally published or not. In 2019, a total of 333 papers were published in the journal, with a median time to first decision of 14 days and a median time from submission to publication of 37 days. The editors would like to express their sincere gratitude to the following reviewers for their generous contribution in 2019:
Aa Haeruman AzamLi SunAbbot O. OghenekaroLiang-Chun WangAbhay PS RathoreLidia Stasiak-RóżańskaAbhijeet A. BakreLiliana Machado R.Ribeiro Da SilvaAbhinav UpadhyayLisa GuardoneAd De GroofLisa M. HarrisonAdewole L. OkunadeLlilianne GangesAgnieszka TorzewskaLorenzo LombardAihua TangLuchang ZhuAine McknightLucía FernándezAkihiko HataLuigi SantacroceAlberto AntonelliLuis Carlos MartínezAlejandro HeuckLuis PiñeiroAleksandra PawlakLukasz KozubowskiAles EichmeierŁukasz M. GrześkowiakAlex Mabou TagneŁukasz StępieńAlexander HerbigM C GjessingAlexander M ShestopalovMª Ángeles Pérez MorenteAlexander MalogolovkinMaciej GliniakAlexander SchoutenMagdalena KaraśAlexandra Ibáñez EscribanoMahmoud A.O. DawoodAlfredo J. GuerraMakoto KurodaAlfredo PalopMałgorzata GieryńskaAline GuidolinMałgorzata ZiarnoAlison Luce-FedrowMalte. KornhuberAlister CraigManfred KöllerAliyar FouladkhahManu De RyckerAlla SplichalovaManuela OliveiraÁlvaro Francisco Lopes De SousaMarc SwidergallAmir S. SirajMarcello Otake SatoAmy L. KlockoMarcello TagliaviaAna Cantos-BarredaMarcelo Sousa SilvaAna Cláudia Correia CoelhoMarcin PigłowskiAna Julia García-MalinisMarcos MancillaAnannya BhattacharyaMarcos Rogério AndréAnca Roseanu ConstantinescuMarcus ScottiAnders JohanssonMaria A. NeginskayaAndrás FodorMaria A. PereiraAndré Luis Costa-Da-SilvaMaria Angeles RojoAndrea LuvisiMaría Auxiliadora Dea-AyuelaAndreana PexaraMaria CaeiroAndreas SuhrbierMaría Dea-AyuelaAndrew M. BormanMaria Diuk-wasserAndrew NelsonMaria Doroteia CamposAndrew W. BrowarMaría Elena González-FandosAngel DesaiMaria Eugenia FranciaAngela BrownMaría Eugenia GonzálezÂngela NovaisMaría Hijosa ValseroAngelina NunziataMaria Isabella PrigigalloAnna Berthold-PlutaMaria Laura RamirezAnna GiammancoMaria Leonor FaleiroAnna Maria VettrainoMaria Luisa TelloAnna Rosa GarbugliaMaria SalvatoAnna Teresa PalamaraMaria Teresa MontagnaAnna ValkoMariana BazAnne SteneMariano Sánchez CrespoAnne-Marie Perchec MerienMaría-Teresa Pérez-GraciaAnnetta ZintlMarilena BazzanoAntoine GessainMarino PrearoAntoine TalarminMario Andres PizzornoAntonello SantiniMárió GajdácsAntonia RojasMarissa Di PietroAntonio José Piantino FerreiraMark A. O’DeaAntonio MastinoMark PeeplesAntonio Rivero-JuárezMark TompkinsApril D. DavisMarkus HoffmannApurba K. BarmanMarta B. LopesArmando Arias EstebanMarta Filipa SimõesArtur AlvesMasoume AmirkhaniArvind SharmaMathieu MateoAsha KumariMatthew G. VargaAshok KumarMatthew S. MacauleyAsja KorajkicMayanka AwasthiBonto FaburayMeera G. NairAtul Kumar SinghMelanie T. CushionAtul SharmaMelina KerouAyush RanawadeMelinda BrindleyBahtiyar YilmazMelissa FernandezBaki AkgülMelissa Garcia-ShermanBarbara GloverMelissa H. YoshimizuBarbara HissaMeriel JonesBarbara S. SchnierleMichael FederleBarbara SchneiderMichael HolbrookBastian KromMichael P. ReichelBei GaoMichael SchreiberBenjamin LampMichael Watson Jr.Bernard DellMichal LinialBernard DelmasMichele TotaroBernardo MainouMichelle VisserBert DevriendtMihaela Ileana IonescuBinod KumarMika TarkkaBiruta BankinaMiloš LukáčBjörn KrenzMing Kun HsiehBlake HildrethMiqdad DhariwalaBonnie WebsterMira EdgertonBoris GorovitsMirja PuolakkainenBradford K. BergesMohan AmarasiriBrandon LuedtkeMonica EmbersBritta MöhlMonika Szymańska-CzerwińskaBruce A. RosaMonish Ram MakenaBruno A.C.M. SantosMrinal BhattacharjeeBruno BagnoliMuhammad SaleemCaitlin MullarkeyMuhammad Sohail ZafarCameron ParsonsMukesh GautamCamila CoelhoMukesh KumarCarla MavianMunegowda KoralurCarlo ContiniNagarjuna Reddy CheemarlaCarlo SalernoNagendran TharmalingamCarlo TicconiNam-Hyuk ChoCarmen ChifiriucNancy LinCarmen SieiroNaphak ModhiranCarolina H. PohlNatasha BotwrightCarolina MehaffyNathan ShererCatarina CamposNeeltje Van DoremalenCatherine EichwaldNeeraj ChauhanCélia F. RodriguesNianshuang WangChandan PalNicholas CianciottoCharles S. AppersonNicholas JohnsonCharlotte HouldcroftNicky BullerChiara PiubelliNicola JonesChih-Jung ChenNiedzielska IwonaChin-Chuan HungNiraj ShilChitra UpadhyayNorbert MüllerChris NethertonNorbert NowotnyChristine UnterwegerNoura DosokyChristopher BroderNuruddin UnchwaniwalaChristopher N. LaRockOhad MazorChristopher V. HughesOrusa RiccardoChukwunonso NzeluPallavi SinghChunfeng LiPalmiro PoltronieriChunliu ZhuoPanagiota XaplanteriCintia Hiromi OkinoPanagiotis KaranisClaire CrossanPaola SperandeoClaudia RückertPaolo PastorinoConstantinos TsioutisPartha KarmakarConsuelo CarrilloPascal Van Der WeeleCristina Howard-VaronaPascale PerrinDagmar RingePatricia GravesDagmar StehlikovaPatricia Resa-InfanteDaisuke SanoPaul KinchingtonDaisuke TakamatsuPaulina Niedźwiedzka-RystwejDalibor SedláčekPaulina Rajko-NenowDaniel CameronPedro SouzaDaniel DepledgePejin BorisDaniel FinePeng XuDaniel KornitzerPeter LipkeDaniel MeltersPeter MayneDaniel O’ConnorPhilip W. WertzDaniel SteinPhilippe ColsonDaniele ProvenzanoPierluigi BonelloDardiotis EfthimiosPietro G. TiscarDariusz MłynskiPinelopi SamaraDavid J. LeaverPio Maria FurneriDavid AllenPiotr SzwedaDavid Cole StevensPoom AdsakwattanaDavid HiggsPravin KaldhoneDavid K. MeyerholzPriscila M Da Silva CastanhaDavid RamiloPriya HalvorsenDawn WeirPruthvi Balachandra KalyandurgDebora DellamariaPrzemyslaw CwynarDéborah MerdaPuck Van KasterenDer-Shan SunQiuyan ShaoDiane ParkR Blake BillmyreDimosthenis ChochlakisRachel A. KatzenellenbogenDipesh DhakalRachel BreytaDirk LangeRaffael NachbagauerDomenico TortorellaRaffaela PeroDonald J. AlcendorRaghavendra YadavalliDonna KoslowskyRahul DuttaDra. Virginia AragonRahul JayantDuc Cuong BuiRakesh GanjiEbtihal AlshabibRandal N. JohnstonEduardo GottuzoRandy AlbrechtEdward R.B. MooreRaquel Muñoz-MorenoEdyta WieczorekRashika EI RidiEeva J. VainioRebecca ChristoffersonEfstathios A. KatsifasReed F. JohnsonEgídio TorradoRelja BeckEike SteinmannReto BrunEinar BirkelandRichard BinghamElena Atrián-BlascoRichard BowenElena CriscuoloRichard DelahayElena GonellaRichard M. BeteckEli DuekerRichard UwieraElizabeth WitherdenRijkelt BeumerElvia Janeth OsorioRinaldo PellicanoEman AnisRitwij KulkarniEmanuel VamanuRobert CrossEmma C HobbsRobert J. BastidasEnrico De LilloRobert WojtyczkaEran BosisRobert Y.-L. WangEric GhigoRodney HoffEric Wellington RiddickRoland ZellErin M. RehrigRomain PaillotEsther E. Biswas-FissRomana VulturarEugene MacCarthyRosa AraujoEva KaclikovaRosa GaglioneEva SapiRositsa Denkova-KostovaEva VarallyayRoss BeierEwa BokRowena BullEwa KaczorekRui M.S. CruzEwa PlantRuiyong ZhangEwelina KrolRupsa DattaFabrizio BertelloniRuth Serra-MorenoFan ZhangRyan RegoFany ReffuveilleSabrina Cadel-SixFarah Asa'adSachio TsuchidaFayna Diaz-San SegundoSae Woong ParkFederica Di ProfioSaguna VermaFeng QuSam AlsfordFernando MonroySam PetersonFernando Pliego-AlfaroSamina AkbarFetweh Al-SaleemSamuel A.M. MartinFilippo FratiniSamuel CollinsFiona M. SansomSamuel K. MutigaFlorian KrammerSandeep UpadhyayFlorin George HorhatSandra Martínez-TuriñoFrancesca ManciantiSara BernardiFrancini KlaumannSara BonettaFrancisco LlorenteSara SulimanFrancisco Ramos MoralesSara TramontiniFranco H. FalconeSarah CaddyFrank PesslerSarah SappFreddy Ibanez CarrascoSarah Sze Wah WongFun-In WangSatheeshkumar PalanisamyGabriela Jorge Da SilvaSatish RainaGabriela MotoieScott BowdenGabriele A. LandoltSebastian GnatGaetano IsolaSelma GiorgioGanyu GuSerena DelbueGavin K. PatersonSergio VargasGema Alama-BermejoSeung-Yoon OhGen KanekoShanmugam MuruganandanGeorge B KyeiShannon RoncaGhasem RashidianShaolei TengGiedrė AlzbutienėSheila CroweGina HarperSherif T.S. HassanGonzalo MoratorioShigeki KamitaniGöran WadellShinji MatsudaGoutam Kumar TantiShreesh SammiGraham NugentShuvo BakarGrant HughesShyamal ChoudhariGraziella BertaSI KawazuGregory CaputoSigrun EickGuillermo Ignacio Perez-PerezSílvia A. SousaGuofang ShenSilvia BonettaHarini IyerSinead M. SmithHassan MahsoubSlavena VylkovaHeba AlzanSmriti VermaHee-Soo ParkSonu SubudhiHelen BuseSophie GranierHelen HuSophie KathariouHélène SanfaçonSophie RodriguesHelle Bielefeldt-OhmannSophie ShawHema Prasad NarraSouleymane DoucoureHercules SakkasSpiros ParamythiotisHirofumi ShimomuraSreekumar OthumpangatHiromi Takahashi-OmoeSrikanth Reddy JangaHironori YoshiyamaSrinath PashikantiHon HoStefan WorgallHua QinStefanie BarbirzHyun-Woo ParkStefano AquaroIgnacio MenaStefano PetriniIgor SplichalStella Maris González CappaIldikó BácskayStella Maris PezzottoIlse JacobsenStephane LemiereImaan BenmerzougaStephen W. TuffsImran SiddiquiStephen ForsytheInga EichhornSteven BradfuteIrina KontsevayaSteven RockmanIsabel Joao Soares SilvaSteven SingerIvan Sanz MuñozSteven Van LanenIvy DambuzaStriker RobJ. Claire HovingSubbulakshmi SureshJacek KaramonSubha DasJack NunbergSudip RayJacob Lorenzo-MoralesSue Ellen CrawfordJacques ThibodeauSujeet KumarJaime CarrascoSukhvinder SinghJakub Dalibor RybkaSumit GhoshJames B. DaleSunil KumarJanetta NiemannSuresh KuchipudiJan-Ulrik DahlSusan CorkJason E. ComerSusan JosephJason KaelberSwinburne A.J. AugustineJason KindrachukSy Duong-QuyJavier Ventura-JuárezSybren G. de. HoogJean A. BernatchezSylvia BruistenJean O’DwyerSylwia AndrzejczukJean-Heinrich DaugroisTahl ZimmermanJeff WiluszTaisun KimJens Andre HammerlTakayuki MurataJens KrethTanel PungaJeongmin SongTatsufumi UsuiJeroen D. LangereisTeiji SawaJessica SpenglerTeng PengJesus RomoTeresa LambeJijun TangTeresa O'MearaJimmy T. EfirdTerry Ann ElseJing JinThamarai JangananJoanna Sztuba-SolinskaTheodore S. KapellosJoel N. MaslowThillaiampalam SivakumarJoel RovnakThomas DandekarJoergen SchlundtTimothy John MahonyJohannes WernerTing ZhangJohn CipolloTodd ReynoldsJohn DartTomas DuffyJonathan YewdellTommaso FelicettiJong Hwan KwakTomohiro IshikawaJoo Hwan NoTomomi NakaharaJooyoun ParkTomomi TakanoJosé BengoecheaTor Atle MoJose Maria SantosTracy NicholsonJose ThekkiniathUlrich DesselbergerJosé TuellsValentina Dell'OsteJoyce WilsonVallì De ReJuan Rodriguez-FloresVan Khanh NguyenJulia RomanoVeeranoot NissapatornJulián Solís García Del PozoVeerupaxagouda PatilJulius LukesVelmurugan BalaramanJunji XingVeronica JarockiKai MatuschewskiVesna KrnjajaKai TangVictor HuberKamila Rybczyńska-TkaczykViktoria RungelrathKamlesh PawarVincent BourretKanakala SurapathruduVincent DelormeKapil ChousalkarVincenzina FuscoKarim EssaniVincenzo CavalieriKarl DrlicaVincenzo LionettiKarthik KamathVinko KrstanovićKatarzyna Otulak-KoziełVirag SharmaKathryn E. ReifVirginie DoceulKatrina BoswardVishal Kishor KhatriKazuhide SatoVishukumar AimaniandaKeiji UedaVitaly GanusovKeith WeaverVittorino NovelloKelli L. BarrVivi MiriagouKelly ElkinsVladimir VimbergKendra AlfsonWalid AzabKen-ichiro MinatoWankun XieKenneth LiegnerWansu ParkKevin SokoloskiWarish AhmedKhaled SultanWei-Chen LinKim BlasdellWeili KongKinga StuperWendy PageKiril M. DimitrovWen-Wei ChangKirsten ParrattWilliam SchwanKirstin RossWioletta Adamus-BiałekKoen K.A. Van RompayWolfram BruneKonstantinos TriantafyllouXaver SewaldKrishnendu KhanXi WangKristina G HultenXian CaoKrisztian MagoriXisha WangKrzysztof SkowronYashar SadighKuldeep GuptaYi-Ning ChenKunfeng SunYoichi FuruyaKun-Hsien TsaiYongjun SuiKyoko Tsukiyama-KoharaYoshihiro SakodaLakshmyya N. KesavaluYosra A. HelmyLara ConsoleYutaka KishidaLarry AndersonYuying LiangLarry SimpsonZachary StrombergLaura GoodmanZhe WangLaura RighettiZhicheng DouLei HuangZigui ChenLeidy LagosZorana KatanićLeyi Wang


